# Exosomal Non-Coding RNAs: Diagnostic, Prognostic and Therapeutic Applications in Cancer

**DOI:** 10.3390/ncrna1010053

**Published:** 2015-06-03

**Authors:** Marc D. Bullock, Andreia M. Silva, Pinar Kanlikilicer-Unaldi, Justyna Filant, Mohammed H. Rashed, Anil K. Sood, Gabriel Lopez-Berestein, George A. Calin

**Affiliations:** 1 Cancer sciences unit, University of Southampton School of Medicine, Southampton, SO16 6YD, UK; 2 Department of Surgery, University Hospital Southampton, Southampton, SO16 6YD, UK; 3 Department of Experimental Therapeutics, The University of Texas MD Anderson Cancer Centre, Houston, TX, 77054, USA; E-Mails: amsilva@mdanderson.org (A.M.S.); pkanlikilicer@mdanderson.org (P.K.-U.); mmrashed@mdanderson.org (M.H.R.); glopez@mdanderson.org (G.L.-B.); gcalin@mdanderson.org (G.A.C.); 4 Instituto de Investigação em Saúde, Universidade do Porto, Porto, 8234150-180, Portugal; 5 INEB—Institute of Biomedical Engineering, Universidade do Porto, Porto, 8234150-180, Portugal; 6 Department of Gynecologic Oncology and Reproductive Medicine Unit 1362, The University of Texas MD Anderson Cancer Center, Houston, TX, 77230 USA; E-Mails: jfilant@mdanderson.org (J.F.); asood@mdanderson.org (A.S.); 7 Department of Pharmacology and Toxicology, Faculty of Pharmacy, The University of Al-Azhar, Cairo, 11754, Egypt; 8 Center for RNA Interference and Non-Coding RNAs, The University of Texas MD Anderson Cancer Center, Houston, TX, 77054, USA; 9 Department of Cancer Biology, The University of Texas MD Anderson Cancer Centre, Houston, TX, 77054, USA

**Keywords:** exosome, non-coding RNA, microRNA, long non-coding RNA, cancer, biomarker, therapy

## Abstract

Non-coding RNAs, such as microRNAs and long non-coding RNAs, are important regulatory molecules which are corrupted in cancer, often in a tissue and stage specific manner. Accumulated data suggests that these promising biomarkers, may also form the basis of novel targeted therapeutic strategies. The role of exosomes in cancer development and metastasis pathways is also increasingly well described. These endosome derived extracellular vesicles which are trafficked horizontally between tumor cells, and vertically between tumor cells and the surrounding microenvironment, carry bioactive cargos, which can reprogram the phenotype of recipient cells with important oncogenic consequences.

Exosomes are enriched with non-coding RNA content. Within exosomes, non-coding RNAs are secreted into the peripheral circulation and other bodily fluids where they are protected from enzymatic degradation by the surrounding phospholipid membrane. Exosomes are therefore a highly promising source of diagnostic and prognostic material in cancer. Furthermore, as exosomes are natural ncRNA carriers, they may be adapted for the purpose of drug delivery by the introduction of exogenous ncRNAs or by manipulating their endogenous ncRNA content. In the current review, we will explore these highly clinically relevant themes by examining the roles of exosomal ncRNAs in cancer diagnostics, prognostics and therapy.

## 1. Introduction

Exosomes are 50–90 nm diameter vesicles, comprising a lipid bilayer and enriched nucleic acid and protein content. They form within multivesicular bodies (MVBs), a specialized compartment of the endosomal pathway and are secreted from cells by fusion of MVBs with the plasma membrane [1].

Exosomes are important vehicles of intracellular communication with diverse physiological functions such as the synaptic transfer of protein and RNA [2,3]. However, in recent times their role in cancer pathogenesis has also attracted attention as it has emerged that exosomal shuttling between cells and the tumor microenvironment may have a profound biological impact promoting tumorigenesis and metastatic progression [4,5,6,7]. These effects are mediated by cytokines, growth factors, proteins and lipids, and by non-coding RNAs (ncRNA) [7].

NcRNAs identified within exosomes, include microRNA (miRNA), long non-coding RNA (lncRNA), small nuclear RNA (snRNA), small nucleolar RNA (snoRNA), long intergenic non-coding RNA (lincRNA), piwi-interacting RNA (piRNA), ribosomal RNA (rRNA) and transfer RNA (tRNA) [8,9]. miRNAs and lncRNAs in particular are powerful regulators of homeostasis and cell signaling pathways, with important roles both in health and disease. Given the intuitive manner in which they may be mimicked or suppressed, they are also attractive vehicles for the development of novel pharmacology [10,11]. However, enthusiasm for RNA based drugs is constrained by recognition that safe and effective mechanisms of delivering therapeutic cargos to target tissues are currently lacking [12]. With the discovery of exosomes, a natural mechanism of non-coding RNA transport, which may potentially circumvent some of the challenges of “pay-load” delivery, interest in this novel but highly clinically relevant field has recently been renewed. Equally, miRNAs and lncRNA in plasma and other bodily fluids have been identified as promising biomarkers of disease leading some to postulate that ncRNAs contained within exosomes may provide an enhanced source of diagnostic and prognostic material in cancer [13,14].

In the current review, we will examine the synthesis of these two rapidly evolving areas of cancer research: The clinical application of ncRNA molecules; and the therapeutic and biomarker potential of exosomes. We will discuss a substantial body of data not previously featured in review format and highlight knowledge gaps which may impede translation to the clinical setting.

## 2. ncRNA and Exosomes: A Novel Therapeutic Strategy

### 2.1. siRNA

The notion that endogenous proteins, and coding and non-coding RNA molecules within exosomes are shuttled between cells, [2] and that the content of exosomes is functional within target cells, has profound implications for the initiation and progression of cancer [6].

Alvarez-Erviti *et al.*, were the first to explore whether this natural cell communication mechanism could be harnessed for therapeutic purposes [15]. Using dendritic cell derived exosomes depleted of T-cell mediated immune activators such as MHC-II and CD86 [16], an exogenous siRNA, administered intravenously was targeted specifically to the brain of C57BL/6 mice. Targeting was achieved by cloning the rabies viral glycoprotein (RVG) which binds acetylcholine receptors and is central nervous system (CNS) specific [17], into Lamp2b, a protein found in abundance at the surface of exosomes [18]. Subsequent expression of this chimeric plasmid in mouse bone marrow-derived dendritic cells produced a source of CNS targeted, exosomal homografts into which short interfering RNAs (siRNAs) could be loaded by electroporation [19].

By demonstrating CNS specific suppression of GAPDH mRNA and BACE1, a protein implicated in the pathogenesis of Alzheimer’s, they showed in principle, that targeted gene silencing can be achieved using exosomes as a delivery vehicle. The group also established the low immuno-stimulatory profile of “self” exosomes, even with repeated administration; they demonstrated that therapeutic exosomes could cross the blood brain barrier; and they established *in vivo* that cellular penetration of siRNA molecules, which is constrained by their negative charge and vulnerability to enzymatic degradation, could be substantially improved by encasing them in a phospholipid capsule [15]. In doing so, the authors of this study addressed many of the challenges previously identified with synthetic nanoparticle delivery vehicles [20,21]. Polyethylenimine (PEI) nanoparticles, liposomes and lipid nanoparticles for example, are all highly promising agents for targeted siRNA delivery, but their translational appeal has been limited to varying degrees by toxicity and immunogenicity issues and other off-target effects [22].

Using similar methodology, the potential for exosomal siRNA based targeting of neuro-degenerative [23] and angiogenesis pathways [24] has been examined, however other than an analysis involving the oncogene MAPK in *ex vivo* human monocytes, cancer orientated studies are absent [25]. This is perhaps surprising given the attention siRNA based cancer therapy has attracted in the recent past [26].

### 2.2. miRNA

Although proof-of-concept was achieved using siRNA, the application of exosomal miRNAs in the context of “gene-therapy” is also increasingly well described.

MiRNAs are a class of small (18–22 nucleotide) highly conserved, non-coding RNA molecules which control whole programs of gene expression through the targeted suppression of mRNA translation [27]. Deregulated miRNA expression has been implicated in every cancer examined to date and is linked to each of the hallmark processes of tumorgenesis, as well as metastasis and chemotherapy resistance pathways [28,29,30]. Inhibiting oncogenic miRNAs (oncomiRs) with anti-sense oligonucleotides(anti-miRs), or mimicking tumor suppressor miRNAs, has therefore long been muted as a potential therapeutic strategy in cancer.

Crucially, exosomal miRNAs secreted by donor cells are taken up by recipient cells and remain functional in both *in vitro* and *in vivo* analyses [6,31].

Ohno and colleagues [32] used transiently transfected human embryonic kidney cells to generate exosomes loaded with let-7a, an important tumor suppressor miRNA which is downregulated in various contexts including breast cancer [33]. A further common aberration in epithelial tumors such as breast cancer, is upregulated cell surface expression of the epidermal growth factor receptor (EGFR) [34]. As the peptide GE11 is a specific EGFR ligand, targeted let-7a delivery to EGFR expressing breast cancer xenografts in mice, was achieved by intravenous administration of miRNA laden exosomes which co-expressed GE11 at their surface. *In-vivo* imaging demonstrated preferential migration of GE11 expressing exosomes to tumor tissue, and *in vitro* studies confirmed that targeted exosomal uptake was inhibited in cells in which EGFR had been knocked down using siRNA. Although xenograft growth was significantly impaired in GE11/let-7a exosome treated mice, providing the first direct evidence of therapeutic utility, the expression of let-7a regulated genes was not reduced compared with controls. Further mechanistic dissection is therefore required to substantiate these findings, characterize these seemingly off-target anti-cancer effects and identify molecular processes in which let-7a may be involved [32].

*In-vivo* delivery of cancer relevant miRNAs using exosomes was also achieved by Momen-Heravi and colleagues, who showed that intravenous administration of unmodified B-cell derived exosomes loaded with miR-155 was associated with significantly elevated miR-155 expression in the liver of miR-knockout mice compared with controls [35]. Although the primary aim of this study was to explore the principle of whether in a therapeutic context, deficient miRNAs could be supplemented using exosomal delivery, consensus suggests their choice of candidate (miR-155) is in fact an oncogene [36,37]. However, using an alternative approach, the group also provided evidence *in vitro* that antisense miR-155 inhibitors (antimiR-155) could be delivered via exosomes to recipient cells, and by demonstrating phenotypic changes specifically in macrophages following exposure to antimiR-155 exosomes, they extended the therapeutic paradigm to include the targeting of key cells within the tumor microenvironment [35]. The tumor microenvironment has attracted particular attention in recent years as recognition of its importance during disease progression has made the development of stroma-targeted therapies an attractive prospect [38,39]. Exosomes derived from stromal myofibroblasts appear to be key mediators of a pro-angiogenic, pro-metastatic TGFβ response in cancer epithelial cells [40]. Equally, exosomes originating from cancer cells trigger reciprocal phenotypic changes in the cells of the tumor microenvironment [41]. It has also emerged that endogenous ncRNAs contained within stroma-derived exosomes trigger a latent anti-viral response in adjacent tumor epithelium, which induces chemoresistance in breast cancer cells [42].

Although the significance of exosomal communication between tumor and its microenvironment has only recently begun to emerge, a number of studies have highlighted the potential benefits of targeting this mechanism for therapeutic purposes: miR-1, which is downregulated in human Glioblastoma Multiforme (GBM), inhibits tumor invasion and angiogenesis *in vivo* and impairs exosome release. miR-1 overexpression causes significant changes in the protein composition of exosomes and is itself secreted by cells into the tumor microenvironment [43].

MiR-21 is a key regulator of cellular phenotype for both tumor cells and cells within tumor associated stroma [44,45,46,47]. Alhasan and colleagues showed that antisense-miRNA assembled in spherical nanoconjugates (SNA) on a gold core but packaged within prostate cancer-derived exosomes, induced miR-21 suppression in recipient cells equivalent to a ≈3000-fold greater concentration of free anti-miR-21 SNA [48]. Other than *in vitro* migratory capacity, which was suppressed by miR-21 inhibition, no functional endpoints were examined or clinical extrapolations made in this study. However, as SNAs are themselves promising therapeutic agents, these data do imply that exosomal delivery vehicles may be used in combination with other technological innovations, to boost the efficiency of RNA interference [48].

The extensive role of miRNAs in chemotherapy resistance pathways, suggests a further therapeutic application for exosomes in cancer [49]. Secreted miRNAs such as miR-221/222 which are highly expressed in tamoxifen resistant breast cancer exosomes compared with wild-type, pass to recipient cells *in vitro* and confer upon them an anti-apoptotic, pro-proliferative phenotype. Crucially, this effect can be reversed by treating resistant cells with exosomes laden with anti-sense miRNA inhibitors [50]. Other exosomal miRNAs implicated in the transfer of therapy resistance in breast cancer are miR-100 and miR-30a [51].

Munoz and colleagues identified that miR-9 upregulation in temozolamide (TMZ) resistant GBM cells is linked to expression of the drug efflux transporter, Multi-drug resistance protein 1 (MDR1). Intriguingly they also demonstrated in co-culture experiments, that anti-miR-9 was transported from mesenchymal stem cells (MSC) to GBM cells via exosomes and that ectopic anti-miR-9 in GBM cells conferred sensitivity to TMZ and suppressed MDR1 expression [52]. Although this study was conducted purely *in vitro*, MSCs have tumor-tropic properties *in vivo* [53] and therefore this approach may provide an alternative mechanism of miRNA delivery to tumor sites.

### 2.3. lncRNA

Intense interest in the non-protein coding genome has recently uncovered an entirely novel class of regulatory non-coding RNA. LncRNAs exceed 200 nucleotides in length and act concurrently with DNA-binding proteins and other elements to epigenetically regulate DNA transcription [54,55,56]. Emerging data suggests that lncRNAs participate in key biological processes in both physiology and disease, and that deregulated lncRNA expression has important consequences during malignant transformation [57].

LncRNAs, having been identified in exosomes [8,9] have also been implicated in TGFβ dependent chemoresistance pathways [58]. Takahashi and colleagues characterized lncRNA expression in extracellular vesicles derived from the Hepatocellular carcinoma HCC cell line HEPG2, pre and post TGFβ treatment. They identified significant differences in lncRNA expression relative to parent cells and found certain candidates including lincRNA-RoR to be further enriched in response to TGFβ. These data support the notion that cellular sorting of lncRNAs into exosomes is highly regulated, and suggest that this process may be disrupted in response to paracrine signals from the tumor microenvironment [58].

Exosomal transfer of extracellular vesicles between HepG2 HCC cells conferred a degree of chemoresistance in response to increasing doses of Sorafenib, Doxorubicin and Camptothecin, and siRNA mediated knockdown of lincRNA-RoR was associated with a significant fall in cell viability. Although this analysis did not contain mechanistic studies, for the first time, an exosomal lncRNA which may be targetable with siRNA has been implicated in the regulation of tumor chemoresistance.

These data suggest the exploitation of exosomes as a natural mechanism of ncRNA transport may provide a promising therapeutic mechanism to target tumor tissue, modulate the tumor microenvironment, or sensitize tumor cells to conventional anti-cancer drugs.

**Figure 1:**
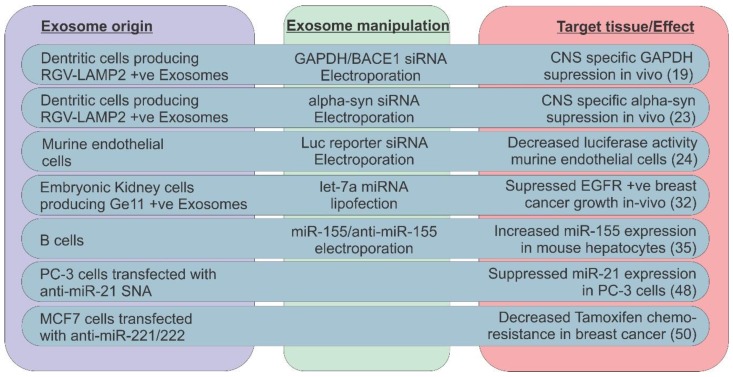
Delivering ncRNA mimics or inhibitors to target cells via exosomes: A putative therapeutic strategy. Exosomes derived from various cell types have been used to deliver tumor suppressor miRNAs (e.g., let-7) or oncogenic miRNA inhibitors (eg anti-miR-21) to recipient cells both *in vitro* and *in vivo*. By manipulating expression of exosome surface markers, organ specific delivery may also be achieved. The delivery of let-7a to EGFR expressing breast cancer xenografts provides the first *in vivo* evidence of an anticancer effect achieved with “therapeutic” ncRNAs packaged in exosomes. Abbreviations: Central Nervous System (CNS); Epidermal Growth Factor Receptor (EGFR); Spherical nanoconjugate (SNA); positive (+ve).

As studies in this field have generally been performed on a small scale, findings are yet to be translated into the clinical setting (Figure 1). In anticipation however, data has emerged exploring the pharmacokinetics of exosomal drug delivery vehicles. In a recent study in mice, unmodified tumor-derived exosomes administered intravenously, were taken up rapidly by liver and spleen but not to a significant extent by tumor tissue [59]. Although intratumoral injection proved a more successful strategy, renal excretion was rapid, illustrating the scale of the bioengineering challenge if exosome based drug delivery systems are to become a realistic prospect.

## 3. Exosomal ncRNAs as Biomarkers in Cancer

Exosomes are considered a highly promising source of potential biomarkers in cancer. They have been isolated from a variety of bodily fluids including blood, urine, saliva, seminal fluid and bronchoalveolar secretions, by non-invasive or minimally invasive means [13,60,61,62,63]. Exosomes are generally increased in the malignant setting and in ovarian tumors for example, exosomal production correlates with tumor stage [64,65]. Furthermore, miRNA loading into exosomes is a tightly regulated process [66] and as exosomal content is protected from enzymatic degradation, miRNA profiles are highly consistent and remain stable even during storage [13,65,67].

Recent stoichiometric analysis suggests that the absolute number of miRNA molecules contained within exosomes is in-fact very low (perhaps less than one miRNA molecule per exosome) [68]. Notwithstanding, accumulated data highlights the potential clinical utility of certain ncRNAs, in particular miRNAs, as diagnostic and prognostic markers of disease.

### 3.1. Diagnostic Markers

Tumor derived exosomes in the peripheral circulation display distinct patterns of miRNA expression compared with exosomes from healthy subjects [65]. As venesection is a well-tolerated, routine clinical procedure, the prospect of detecting early malignant changes using a simple blood-test, is extremely attractive. As with any screening tool however, the sensitivity and specificity of the test is key to clinical translation, but the fact that exosomal miRNAs are protected within a lipid bilayer, whereas freely circulating miRNAs are not, suggests exosomes may be the optimal source for biomarker detection [13,69].

In an early study, Taylor and colleagues identified a panel of eight miRNA candidates which were closely correlated in primary tumor tissue and circulating exosomes from 50 women with ovarian cancer, and which were significantly overexpressed compared with healthy control subjects. This implies that exosomal miRNAs may have some utility as diagnostic biomarkers in ovarian cancer [65].

MiRNA-21 is approximately 40-fold overexpressed in exosomes from patients with GBM compared with normal control subjects [6]. PCR-Array based screening also identified miR-320 and miR-574-3p as potential exosomal markers of GBM diagnosis (ROC curve analysis: miR-320 AUC = 0.719, *p* = 0.0067; miR574-3p AUC = 0.738, p=0.0055); however in an independent validation cohort, no significant differences in expression were noted between patients and matched normal controls [70].

In colorectal cancer (CRC) miR-21 and seven other candidates were differentially expressed in serum exosomes in patients compared with matched healthy control subjects. In terms of diagnostic utility the best performing exosomal miRNAs identified in this study were miR-1246 and miR-23a (ROC curve analysis AUC = 0.948 and 0.953 respectively). Results were validated in an independent patient cohort and interestingly expression of all candidates fell significantly following tumor resection [71]. However, ROC curve analysis for miR-21 (AUC = 0.798) did not compare favorably to an independent analysis of freely circulating serum miR-21 (AUC = 0.919) [72] implying that protection within a phospholipid bilayer may not in fact enhance the diagnostic accuracy of miRNAs.

In a study comparing serum derived exosomal miRNAs in patients with laryngeal squamous cell carcinoma (SCC) and matched patients with benign vocal cord polyps, miR-21 again was shown to have some diagnostic utility (ROC curve analysis: AUC = 0.801: 95%CI 0.710-0.874, *p* < 0.000). However, perhaps the most interesting element of this study was that for the first time, the biomarker potential of an exosomal lncRNA was also examined [73]. HOTAIR is a lncRNA which interacts with the Polycomb Repressive Complex to suppress target gene expression [74]. It has been implicated in the pathogenesis of numerous malignancies [10]. Alone, exosomal HOTAIR expression was capable of distinguishing benign from malignant disease with a sensitivity of 92.3% and specificity of 57.2%, but in combination with miR-21 this was improved to 94.2% and 73.5% respectively [73].

In other studies, exosomes containing miRNA cargo have been identified in serum from patients with metastatic uveal melanoma and lung cancer [64,75]. In lung cancer, an alternative approach has also been used: Rodriguez and colleagues examined serum specimens and upper respiratory tract secretions by bronchoealveolar lavage (BAL) from 30 patients with a diagnosis of lung cancer and 75 patients with non-malignant lung pathology. Although the level of exosomes isolated was significantly greater in serum than BAL, BAL specimens from tumor patients were enriched with exosomes compared with non-tumor patients. Several key regulatory miRNAs including miR-21, let-7b and miR-200 were expressed in BAL tumor exosomes, however a unique tumor profile was not evident [62].

Similarly, exosomal harvest can be achieved non-invasively from cervicovaginal lavage specimens. By this method miR-21 was again shown to be overexpressed in cancer derived exosomes as well as exosomes from patients with human papilloma virus (HPV) positive serology compared with matched control subjects. This result was mirrored for miR-146a, another factor implicated alongside miR-21 and HPV infection in the pathogenesis of cervical cancer [76].

### 3.2. Prognostic Markers

In a recent study by Wang and colleagues, exosomal HOTAIR and miR-21 expression in combination was used to distinguish patients with laryngeal SCC from patients with a non-malignant vocal cord pathology. Intriguingly, expression of both elements also correlated significantly with progressive tumor stage, implying serum derived exosomal ncRNAs may also have utility as prognostic markers in cancer [73].

Three further studies have examined the prognostic potential of exosomal ncRNA molecules: In prostate cancer, miR-375 and miR-1290 expression in serum derived exosomes both correlated significantly with overall survival in a cohort of consecutive patients with castration-resistant disease. When used in combination, patients expressing high levels of both exosomal miRNAs had a mortality rate of 80% at 20 months compared with 10% in the group expressing low levels of both miRNAs. For comparison, median survival was 7.23 months and 19.3 months respectively (p=0.0045) [77]. In breast cancer, exosomal miR-373 expression was elevated in triple negative disease compared with luminal tumors and in oestrogen and progesterone receptor negative breast cancers compared with hormone receptor positive control subjects. These data, which suggest miR-373 expression is associated with a more aggressive breast cancer phenotype, may also help guide decision making in the early phases post-diagnosis [78].

Finally, Sugimachi and colleagues used high through-put miRNA analysis to characterize exosomal miRNAs from serum in HCC patients with and without disease recurrence following liver transplant surgery. In the training set, two candidates (miR-718 and miR-1246) were identified as potential biomarkers of disease recurrence. Although no significant differences were identified in a validation set of 59 consecutive patients, miR-718 expression in combination with other clinicopathological parameters was significantly associated with recurrence free survival [79].

Table 1 summarizes key data published to date in the field of exosomal ncRNAs as cancer biomarkers. However, two further highly relevant studies should also be considered. Firstly, Melo and colleagues demonstrated that miRNA biogenesis may occur within exosomes in a cell independent manner. That is to say that exosomes, which are known to contain Dicer, and AGO2 and TRBP proteins, can propel precursor miRNAs towards maturity even after secretion from the cell. This suggests that miRNA content within exosomes is subject to dynamic change and may not provide consistent expression profiles over time [4]. Notwithstanding, a study by Palma *et al.*, [80] identified that not only are exosomal miRNAs selectively secreted from malignant cells, but the character of exosomes themselves is altered in the malignant setting. There may then be a correlation between exosomal content and exosomal sub-type which can be exploited in the clinical setting to provide disease specific diagnostic or prognostic information.

In summary, the identification of biomarkers, which help predict the onset of disease recurrence or response to therapy, is an important clinical priority. Exosomal ncRNAs are a readily accessible source of cancer relevant biological information, and in the age of personalised therapy, any biomarker discovery strategy with the potential to help optimize drug choices or better define outcomes for individual patients, should be pursued. However, studies currently available in this field are limited by low patient numbers and/or retrospective study design. Further prospective, large-scale and randomized studies are required to better define the potential roles of exosome derived ncRNAs are diagnostic or prognostic markers in cancer.

## 4. Concluding Remarks

The current review provides the most up-to-date appraisal of the clinical roles of exosomal ncRNA molecules in cancer. 

Although a relatively recent development, harnessing the therapeutic potential of exosomes promises to circumvent many of the difficulties identified with synthetic drug delivery vehicles in the past. Modifying the expression of receptor ligands at the exosome surface is potentially an elegant solution to the challenge of providing tissue/cancer specific drug delivery. Furthermore, loading exosomes with “therapeutic cargo” consisting of ncRNAs is consistent with the modern aspiration of providing targeted therapies which are also better adapted to each individual patient’s needs.

Endogenous ncRNAs within circulating exosomes may also be a source of valuable information to help guide therapy decisions and stratify risk in the clinical setting. All evidence suggests that further research in this novel field is likely to yield highly clinically translatable knowledge, with the potential to impact positively on the lives of patients with cancer.

**Table 1:** The biomarker potential of exosomal non-coding RNA in cancer.

Study	System	Source of Exosomes	ncRNA Candidates	Biomarker Potential
Taylor DD *et al.*, [65]	Ovarian cancer	Serum	miR-21; miR-141; miR-200a; miR-200b; miR-200c; miR-203; miR-205; miR-214	Exosomal miRNAs distinguish benign from malignant disease
Skog J *et al.*, [6]	GBM	Serum	miR-21	Differential miRNA expression in serum exosomes from GBM patients *vs.* normal control subjects
Manterola *et al.*, [70]	GBM	Serum	miR-320; miR-574-3p	Exosomal miRNAs may distinguish patients with GBM from normal control subjects
Ogata-Kawata *et al.*, [71]	CRC	Serum	let-7a, miR-1229, miR-1246, miR-150, miR-21, miR-223, and miR-23a	Exosomal miRNAs distinguish patients with CRC from normal control subjects
Wang J *et al.*, [73]	Laryngeal SCC	Serum	miR-21; HOTAIR	Exosomal ncRNAs distinguish benign from malignant laryngeal disease. Small but statistically significant differences in expression observed in tumors of progressive pathological stage
Rodriguez M *et al.*, [62]	Non-Small cell lung cancer	Serum	miR-16; miR-20a; miR-195; let-7a; miR-223; miR-21; let-7b; miR-106b; miR-92a; let-7g	Close homology in exosomal miRNA content in serum and bronchial secretions; and between samples from patients with benign and malignant lung pathology.
		Bronchial secretions	miR-21; let-7b; miR-200c; miR-92a; miR-222; miR-26a; miR-141; let-7a; miR-29c; miR-24	
Liu J *et al.*, [76]	Cervical cancer	Cervicovaginal secretions	miR-21; miR-146a	Exosomal miRNA candidates elevated in cervical cancer compared with healthy HPV +ve and −ve control subjects
Huang X *et al.*, [77]	Prostate cancer	Serum	miR-1290; miR-375	Exosomal miRNAs associated with poor overall survival in castration resistant disease
Eichelser, C *et al.*, [78]	Breast cancer	Serum	miR-373	Exosomal miRNA associated with more aggressive breast cancer phenotypes
Sugimachi *et al.*, [79]	HCC	Serum	miR-718; miR-1246	Exosomal miRNA expression in combination with other clinicopathological parameters associated with progression free survival
